# Investigating the association between African spontaneously fermented dairy products, faecal carriage of *Streptococcus infantarius* subsp. *infantarius* and colorectal adenocarcinoma in Kenya

**DOI:** 10.1016/j.actatropica.2017.10.018

**Published:** 2018-02

**Authors:** Dasel W.M. Kaindi, Wambui Kogi-Makau, Godfrey N. Lule, Bernd Kreikemeyer, Pierre Renault, Bassirou Bonfoh, Esther Schelling, Jakob Zinsstag, Christophe Lacroix, Leo Meile, Christoph Jans, Jan Hattendorf

**Affiliations:** a Department of Food Science, Nutrition and Technology, University of Nairobi, P. O. Box 29053 - 00625, Nairobi, Kenya; b School of Medicine, University of Nairobi, P. O. Box 19676, Nairobi, Kenya; c Institute of Medical Microbiology, Virology, Hygiene and Bacteriology, Rostock University Medical Center Rostock, Schillingallee 70, 18055 Rostock, Germany; d Micalis Institute, INRA, AgroParisTech, Université Paris-Saclay, 78350 Jouy-en-Josas, France; e Centre Suisse de Recherches Scientifiques en Côte d’Ivoire (CSRS), Km 17, Adiopodoumé, Rte Dabou, 01 BP 1303 Abidjan 01, Cote d’Ivoire; f Department of Epidemiology and Public Health, Swiss Tropical and Public Health Institute, Socinstrasse 57, 4002 Basel, Switzerland; g University of Basel, Petersplatz 1, 4003 Basel, Switzerland; h Laboratory of Food Biotechnology, Institute of Food Nutrition and Health, Department of Health Science and Technology, ETH Zurich, LFV C22, Schmelzbergstrasse 7, 8092 Zurich, Switzerland

**Keywords:** cFPD, commercial fermented dairy products, CRC, Colorectal cancer, tFDP, Traditional fermented dairy products, GIT, Gastrointestinal tract, KNH, Kenyatta National Hospital, OR^A^, Odds ratio adjusted, SBSEC, *Streptococcus bovis*/*Streptococcus equinus* complex, *Sgg*, *Streptococcus gallolyticus* subsp. *gallolyticus*, *Sgm*, *Streptococcus gallolyticus* subsp. *macedonicus*, *Sgp*, *Streptococcus gallolyticus* subsp. *pasteurianus*, *Sii*, *Streptococcus infantarius* subsp. *infantarius*, yFPD, yoghurt fermented dairy product, Spontaneously fermented dairy products, Colonic disorders, *Streptococcus bovis*, *Streptococcus gallolyticus*, Colitis, Colorectal cancer

## Abstract

Consumption of traditional fermented dairy products (tFDP) in Africa leads to the ingestion of up to 10^8^*Streptococcus infantarius* subspecies *infantarius (Sii)* per millilitre of spontaneously fermented milk*. Sii* is a member of the *Streptococcus bovis/Streptococcus equinus* complex (SBSEC) for which some members are associated particularly with colorectal cancer or endocarditis. The extent of health risks to tFDP consumers is largely unknown.

A hospital-based unmatched case-control study was conducted at Kenyatta National Hospital, Nairobi (Kenya) on 80 cases and 193 controls that were selected exhaustively from patients attending colonoscopy at the hospital. Logistic regression models adjusted for age, sex and residency were used in the statistical analysis. Consumption of tFDP was not associated with CRC (odds ratio (OR) 1.4; 95% Confidence interval (CI) 0.7–2.7; p = 0.34). Risk factors associated with CRC included age above 40 years, and consumption of processed meat and alcohol. Faecal carriage of *Sii* was significantly higher in persons with colon tumours and polyps compared to controls (8.4% vs 21.6%: OR: 4.6; CI 1.3–15.9). Patients with haemorrhoids represented an unexpected carrier group with significantly higher *Sii* faecal carriage (30.4%, CI: 17.7–45.8). Consumption of tFDP does not represent risk factors for CRC whereas *Sii* seems to be associated with CRC. However, there is urgent need to assess this finding also in the general population, investigate the causality of SBSEC, *Sii* and CRC as well as compare the phylogenetic, functional and genomic relationship between human and dairy *Sii* with regards to the ongoing application of *Sii* in FDP production.

## Highlights

• Study enrolled 449 participants: 193 normal colon & 80 colorectal cancer/polyp cases.
• Overall *S. infantarius* prevalence of 17.3% (34 out of 196 participants).
• Sig. lower *S. infantarius* prevalence in control vs case groups (8.4%, vs 21.6%).
• Significantly higher *S. bovis* complex member prevalence in patients with haemorrhoids.
• Investigation of disease causality and *S. infantarius* prevalence in population needed.

## Graphical abstract

**Figure:**
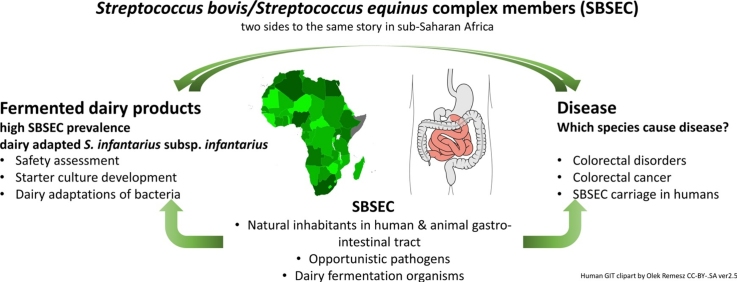

## 1 Introduction

Globally, colorectal cancer (CRC) is the third most common cancer among men and second among women (International Agency for Research on Cancer, 2012) with nearly 1.4 million new cases diagnosed in 2012 (World Cancer Research Fund, 2016). In Kenya, CRC ranks 7th for cancers in both sexes with a cumulative incidence of 4.2 per 100,000 persons per year (International Agency for Research on Cancer, 2012, Korir et al., 2015). CRC is associated with a number of environmental factors including: age above 50 years, smoking, drinking alcohol, obesity, consumption of saturated fats and red meat (Joint WHO/FAO expert consultation group, 2003). Microorganisms such as members of the *Streptococcus bovis/Streptococcus equinus* complex (SBSEC) have also been associated with CRC (Ben-Chetrit et al., 2017, Boleij and Tjalsma, 2013, Corredoira et al., 2015). Despite this association, some SBSEC members are also given commensal status in the gastrointestinal tract of humans and animals, while recent studies have even shown that traditional fermented dairy products (tFDP) in Africa are predominated by *Streptococcus infantarius* subsp. *infantarius* (*Sii*) and to a lesser extent by *Streptococcus gallolyticus* subsp. *macedonicus* (*Sgm*) (Jans et al., 2013b) with up to 10^8^ live *Sii* per millilitre of tFDP (Abdelgadir et al., 2008, Jans et al., 2012a). *Sii* and *Sgm* are members of the SBSEC (Jans et al., 2015, Schlegel et al., 2003). The SBSEC and CRC correlation was originally associated to *S. bovis* (Klein et al., 1977). Advancing taxonomic differentiation specified this association mainly to *Streptococcus gallolyticus* subsp. *gallolyticus* (*Sgg*) formerly classified as *S. bovis* biotype I and *Streptococcus gallolyticus* subsp*. pasteurianus* (*Sgp*), formerly biotype II.2 (Ben-Chetrit et al., 2017, Jans et al., 2015). However, these advances in taxonomic differentiation among the SBSEC opened questions on disease associations of the *S. infantarius* branch (biotype II.1) including *Sii* and *Streptococcus lutetiensis* (intermediately named *Streptococcus infantarius* subsp. *coli*). Disease association with infective endocarditis and other clinical syndromes was partially clarified for *S. lutetiensis* but not for *Sii* as accurate epidemiological data on *Sii* is scarce (Ben-Chetrit et al., 2017, Jans et al., 2015, Romero et al., 2011). In parallel to taxonomic advances, some *Sii* strains have been reclassified as *Streptococcus equinus* while others implicated as human or livestock pathogens were confirmed to be *Sii* suggesting the need to investigate the role of *Sii* with respect to public health (Jans et al., 2016). This is particularly important for the sub Saharan African setting where exposure of humans to live *Sii* through food such as tFDP is high in multiple countries (Abdelgadir et al., 2008, Jans et al., 2012a, Jans et al., 2016, Jans et al., 2013b, Wullschleger et al., 2013).

The long tradition and wide distribution of tFDPs as food products in sub-Saharan Africa reflects the important role of tFDP in nutrition, food safety and food security for communities in this region (Franz et al., 2014, Jans et al., 2017). Considering the large population consuming and relying on tFDP as part of their diet, the elucidation of the role of *Sii* is pivotal. Comparative genomics and phylogenetic analyses of *Sii* strains indicated two main African *Sii* lineages with a rather recent and currently ongoing process of dairy adaptation (Jans et al., 2016, Jans et al., 2013a, Jans et al., 2012b). The dairy adaptations of *Sii* lineages seem to parallel the evolution of *Streptococcus thermophilus* as starter culture in Western fermented dairy products and suggest an important technological role during tFDP manufacturing (Jans et al., 2013a). In this context, African variants of *Sii* have been suggested for evaluation as indigenous starter culture for African tFDP (Jans et al., 2016, Jans et al., 2013a). Therefore, a thorough safety assessment of *Sii* for any future product development and estimation of its public health risk is necessary. However, basic appropriate epidemiological data on *Sii* in Africa and even worldwide is currently lacking.

To provide the first epidemiologic data on CRC, tFDP and *Sii,* we implemented a hospital-based study and enrolled patients at the endoscopy unit of Kenyatta National Hospital (KNH) in Nairobi, Kenya, a country with well-characterized *Sii*-containing tFDP. The main objectives of this study were: i) assessing the relationship between the consumption of tFDP and colon-related health conditions, ii) determining the prevalence of *Sii* in patients with different colon-related health conditions, and iii) identifying additional environmental risk factors such as consumption of other food products, smoking, drinking alcohol, obesity and level of physical activity.

## 2 Materials and methods

### 2.1 Study design

#### 2.1.1 Sample size determination of participants and dairy diet

The anticipated sample size of individuals involved as study subjects was determined assuming a proportion exposed to the consumption of tFDP of 60% (Jans et al., 2013b) among controls and 75% among cases and a case/control ratio of 1:3. Applying Fleiss’ formula for unmatched case control studies, 104 cases and 310 controls are required to detect a statistically significant difference with a power of 80% at the 95% level of confidence. Concerning the dairy diet, it was distinguished between (1) traditional FDP (tFDP) from spontaneously fermented raw milk including informal sour milk (tFDP1) and home-made sour milk (tFDP2); (2) sour milk from heat-treated milk and subsequently fermented with commercial non-*Sii*-starter cultures (cFDP), often named mala/lala; (3) Western-type yoghurt with light/medium to firm consistency made from heat-treated milk by commercial starter cultures containing *S. thermophilus* (yFDP); and (4) cereal-based porridge with either type cFDP or tFDP before cooking the porridge.

#### 2.1.2 Case definition, inclusion and exclusion criteria

**Fig. 1:**
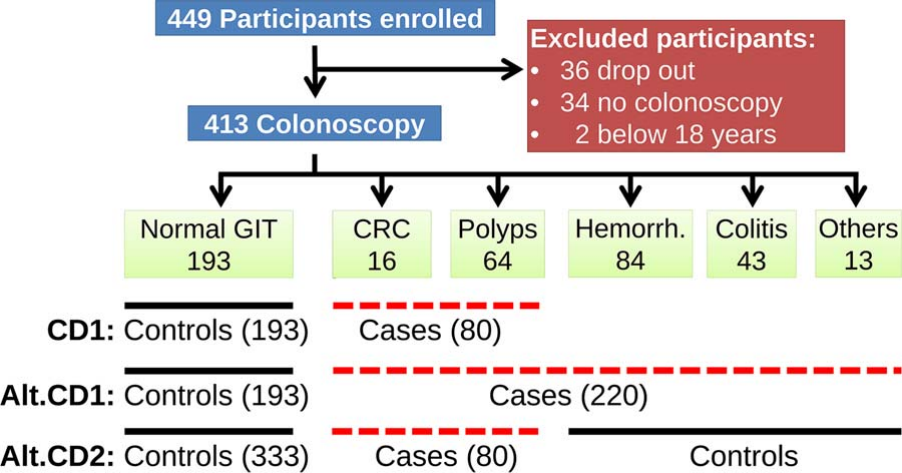
Data flow diagram of participant recruitment among endoscopy patients at Kenyatta National Hospital, stratification by endoscopy examination outcome and case definition (CD) to determine colorectal cancer (CRC), *Sii*/SBSEC and FDP association. Alternative case definitions (Alt.CD) were formulated for sensitivity analysis.

Participants were stratified by the outcome of the colonoscopy to either patients with normal colon (including minor complications such as constipation, abdominal pains or helminths), CRC, adenocarcinoma (polyps), colitis, haemorrhoids or other. A case was defined as an individual whose diagnosis showed colon tumours or polyps while controls were defined as individuals with normal colon. To increase statistical power and to prove that the results are not sensitive to the case definition, two alternative definitions were considered by including patients with colitis, haemorrhoids or other conditions once in the case and once in the control group (Fig. 1). The inclusion criteria were persons of 18 years and above referred for colonoscopy examination. Participants matching the following exclusion criteria were not considered for the study: undertaking chemotherapy, radiotherapy or surgery and pregnancy. Age was self-reported by participants or derived from their date of birth where available.

#### 2.1.3 Participant enrolment and interview

All adult individuals attending KNH for colon examination by colonoscopy at the Endoscopy unit were invited to participate. They were briefed on the study goals and risks, according to the ethical guidelines including the signing of a consent form for voluntary study participation. This was then followed by interviewer-guided filling of the questionnaire, a process that took approximately 30 min. Questionnaires were administered in English, Swahili or local dialect with the help of a translator if needed.

#### 2.1.4 Clinical specimen collection

The colonoscopy revealed the colon health status while microbiological analysis on the clinical samples allowed for detection and isolation of *Sii* and SBSEC. All clinical specimens were collected by a registered nurse or doctor (gastroenterologist) of KNH. Blood sample (4.5 mL) and a rectal swab were obtained before colonoscopy. Blood sample collection followed the international standards (World Health Organization, 2010). The collection of rectal swabs was performed by the medical staff before standard colonoscopy procedure was performed. A sterile swab was used after the anal area was cleaned with 70% ethanol to reduce exterior microorganisms. The swab was inserted into the rectum, rotated gently and removed. The swab was then replaced into the sterile holder until streaking on the culture media the same day. Faecal sample collection was planned in such a way not to interfere with the routine colonoscopy preparation procedures of KNH. Faecal sample collection was therefore performed at home level by the participant prior to self-administration of laxatives the day before colonoscopy. The sterile single-use stool collection tube was issued during the initial participant recruitment. CRC or polyp or tissue samples were collected and screened for SBSEC bacteria when such a tissue sample was taken by the physician for CRC screening. Initial microbiological analyses of all samples were performed at the same day by the in-house bacteriology laboratory of KNH. Histology examination of suspected cancerous tissue samples from the colon of patients was differentiated as follows: malignant lesions/carcinoma were summarized under the term “tumour”. Histology did however not differentiate between neoplastic and non-neoplastic polyp types. The class “polyps” therefore summarizes both types. According to the initially formulated case definition, patients with tumours or polyps were considered as cases.

#### 2.1.5 Ethical considerations and approvals for the study

This study was approved in Kenya by the Kenyatta National Hospital/University of Nairobi Ethics and Research Committee approval number P389/07/2012. In Switzerland, Ethics Committees of ETH Zurich and Kantonale Ethik Kommission Zurich approved the study under decision numbers EK 2013-N-78 and KEK-StV-Nr. 47/14, respectively. The study participants gave consent during enrolment to the study. The study was conducted in accordance with the Declaration of Helsinki (World Medical Association, 2013).

### 2.2 Data collection tools and variables

A structured questionnaire was designed to capture various variables including demographic and socio-economic status, colonic disorders, co-morbidities, physical activity, familial history of CRC, height, weight and food consumption. A check list for clinical samples that included rectal swab, faeces, blood and tumour/polyp tissue was included.

### 2.3 Microbiological analysis of human clinical samples and isolation of presumptive SBSEC strains

#### 2.3.1 Reference strains

Reference strains were obtained from the culture collections of the Laboratory of Food Biotechnology of ETH Zurich (LFBT, Zurich, Switzerland), Culture Collection of the University of Gothenburg (CCUG, Gothenburg, Sweden) and the Deutsche Stammsammlung für Mikroorganismen und Zellkulturen GmbH (DSMZ, Braunschweig, Germany). All analyses were performed using *Sii* CJ18 (Jans et al., 2012b), *Sii* CCUG43820^T^, *Sgg* DSM16831^T^, *Streptococcus thermophilus* DSM20259, and *Enterococcus faecium* DSM20477^T^ as reference strains.

#### 2.3.2 Growth media, supplements and chemical reagents

KF *Streptococcus* (KFS, Becton Dickinson, Allschwil, Switzerland), Mitis Salivarius (MS, Becton Dickinson), Brain Heart Infusion (BHI, Biolife, Milan, Italy) and Trypton Soy Broth (Sigma-Aldrich, Buchs, Switzerland) including corresponding supplements were prepared according to manufacturers’ instructions. All chemicals were obtained from Sigma-Aldrich (Buchs, Switzerland) unless noted otherwise.

#### 2.3.3 Microbiologic analysis of human clinical samples

The blood sample was transferred into universal biphasic glass culture bottles (Biomerieux, France) containing sterile Tryptone Soy Broth (Sigma-Aldrich) by injecting through the rubber stopper. Blood cultures were incubated at 37 °C without agitation. Visual examination of blood cultures was performed within 24 h and at daily intervals thereafter for 5–7 days. Visual evidence of microbial growth included turbidity, haemolysis, gas production or formed colonies in the undisturbed sedimented blood.

Rectal swabs were streaked on KFS (Becton Dickinson) and MS (Becton Dickinson) agar media by rubbing the swab on the agar surface. The swab was then dipped in saline solution and streaked onto a 2nd agar medium of both agar types as a secondary cultivation mean. Stool samples were serially diluted to 10^−3^. Subsequently, approximately 15 μL of each stool sample dilution as well as the pure stool sample were streaked onto KFS and MS agar media using a sterile inoculation loop. KFS and MS agar media were incubated at 37 °C for 24–48 h under aerobic conditions to isolate facultative anaerobe *Sii*/SBSEC and suppress strict anaerobe members of the gut microbiota.

#### 2.3.4 Macro-morphological characterization and isolation of presumptive SBSEC members

After incubation, qualitative description of colonies on KFS and MS agar media was performed by describing their size, colour, edge and surface texture. Two colonies of each morphology type were isolated per medium per participant and streak-purified on the corresponding medium. Each isolate was thereby traceable back to its origin. Purified stock cultures were then stored in BHI broth containing 33% (v/v) glycerol.

#### 2.3.5 Identification of presumptive SBSEC members by molecular tools

DNA of purified strains was extracted and subsequently used in rep-PCR fingerprinting, SBSEC-specific 16S rRNA gene PCR-assay and partial *groEL* amplification and sequencing as previously described (Jans et al., 2016, Jans et al., 2013b, Jans et al., 2012c). All primers for these three PCR assays and sequencing were obtained from Microsynth (Balgach, Switzerland). PCR Master Mix 2 × concentrated (Thermo Scientific, St. Leon-Rot, Germany) was used for all assays. Sanger sequencing of purified DNA amplicons was performed at GATC using the individual primers from the corresponding PCR assay (Konstanz, Germany).

#### 2.3.6 Identification of non-SBSEC isolates by 16S rRNA gene sequencing

To elucidate the species status of bacterial isolates not identified as SBSEC members, a universal 16S rRNA gene PCR assay using bak4 and bak11 w primers was performed (Amann et al., 1990, Greisen et al., 1994). For that, 30 isolates originating from 25 participants representing 30 different rep fingerprints were selected among the overall non-SBSEC isolate pool consisting in 1356 isolates. Amplified DNA fragments of the 16S rRNA gene were then sequenced at Microsynth (Switzerland) using bak11 w as Sanger sequencing primer.

### 2.4 Statistical data analysis

The database was created in Microsoft Access (Microsoft Corporation, Redmond, WA, USA) and statistical data analysis conducted using Stata version 14 (Stata Corporation, College Station, TX, USA, 1984–2000). Dietary intake variables assessed by a semi-quantitative food frequency questionnaire were analysed by using logistic regression adjusted for age group (<40, 41–60 and >60yrs), gender and rural residency. Odds ratios (OR) with their corresponding 95% confidence intervals (CI) were used to measure strength of associations with P-values of less than 0.05 considered statistically significant. All DNA sequence analyses were performed in CLC Genomics Workbench (version 7.5, Qiagen Aarhus A/S, Denmark). Raw sequence chromatograms were trimmed for quality using a minimum stringency setting of 0.01. Trimmed sequences were then queried against the GenBank database using NCBI Blast algorithm to obtain the most likely species identification.

## 3 Results

In total, 449 participants were enrolled in the study out of which 34 did not complete colonoscopy and an additional two were excluded because of age below 18 to arrive a sample size of 413 participants (Fig. 1). To allow for designing the case and control groups, the participants were classified depending on their colon health status, resulting in 193 (46.7%) normal colon, 16 (3.8%) colon with tumour (carcinoma), 64 (15.5%) polyps (non-neoplastic and neoplastic/adenoma), 84 (20.8%) haemorrhoids, 43 (10.4%) colitis and 13 (3.1%) other conditions. Therefore, this study is based on case definition one (Fig. 1) that includes colon with tumour (carcinoma) or polyps (non-neoplastic and neoplastic/adenoma) as the case group (80 participants) while the control group includes participants with normal colon (193 participants), to yield the study population of 273 for the case-control study. However, clinical samples were available from 196 participants.

### 3.1 Demographic characteristics of the study population

The mean age among cases was 54 years (standard deviation, sd 16) compared to 46 years (sd 14) among controls. In the control group, 51% of the participants were male compared to 59% in the case group (Table 1). About 65% of the cases had an educational level above primary school but only 49% of the controls. For about half of the patients the reason for referral was available. The most common reason was rectal bleeding (36% cases, 30% controls) followed by chronic constipation (13% cases, 30% controls). First and second degree of familial history of colorectal cancer was not assessed for association with CRC/polyps, due to low numbers.

**Table 1:** Socio-economic, demographic symptomatology traits in association with CRC of the study population.

N = 273		Normal GIT		CRC		OR	95% CI	P-value
		n = 193	%	n = 80	%			
**Age**								
18 to 30		28	14.5	9	11.3	1.4^a^	1.2–1.5	0.001
31 to 40		42	21.8	6	7.5			
41 to 50		45	23.3	15	18.8			
51 to 60		44	22.8	16	20.0			
61 to 70		18	9.3	19	23.8			
over 70		16	8.3	15	18.8			
age over50		78	40.4	50	62.5	2.5	1.4–4.2	0.001
**Gender**								
Male		100	51.8	47	58.8	–		
Female		93	48.2	33	41.3	0.8	0.4–1.3	0.30
**Education**								
Did not go to school		10	5.2	9	11.4	–		
Primary		58	30.2	31	39.2	0.6	0.2–1.6	0.31
Secondary		73	38.0	23	29.1	0.4	0.1–1.0	0.04
Tertiary		43	22.4	9	11.4	0.2	0.1–0.7	0.01
University		8	4.2	7	8.9	1.0	0.3–3.8	0.97
**Household size**								
One		16	8.3	5	6.3	–		
2–4 including children		59	30.6	19	23.8	1.0	0.3–3.2	1.0
4–6 including children		34	17.6	12	15.0	1.1	0.3–3.8	0.8
> 6 including children		17	8.8	12	15.0	2.3	0.6–7.9	0.2
**Marital status**								
Single		48	24.9	14	17.5	–		
Married		139	72.0	61	76.3	1.5	0.7–2.9	0.23
Separated		6	3.1	4	5.0	2.3	0.6–9.2	0.25
**Familial history for CRC**								
1st degree relatives		7	3.6	2	2.5	nd^b^		
2nd degree relatives		7	3.6	3	3.7	nd		
**Residence**								
Urban residence		107	55.4	35	43.8	–b		
Rural residence		86	44.6	45	56.3	1.6	1.0–2.7	0.08
**Own livestock**								
No		71	36.8	21	26.3	–		
Yes		52	26.9	26	32.5	1.7	0.9–3.3	0.13
**Contact with livestock**								
No		178	92.2	70	87.5	–		
Yes		15	7.8	10	12.5	1.9	0.8–4.6	0.15
**Occupation**								
Dependent		45	23.3	21	26.3	–		
Employed		62	32.1	20	25.0	0.7	0.3–1.4	0.32
Business person		46	23.8	14	17.5	0.7	0.3–1.4	0.29
Self-employed		21	10.9	12	15.0	1.2	0.5–2.9	0.65
Casual labourer		16	8.3	13	16.3	1.7	0.7–4.3	0.23
**Roof Material**								
Iron sheets		134	69.4	57	70.0	–		
Roofing tiles		11	5.7	11	13.8	2.3	1.0–5.8	0.06
Grass thatched		1	0.5	1	1.3	2.4	0.1–38.9	0.54
Concrete		44	22.8	11	13.8	0.6	0.3–1.2	0.17
**Source of drinking water**								
Tap water		139	72.0	55	68.8	–		
Borehole water		31	16.1	12	15.0	1.0	0.5–2.0	0.95
Rain water/pond^c^		23	11.9	13	16.3	1.4	0.7–3.0	0.35
**BMI**								
Underweight		15	7.8	8	10.0	0.9^a^	0.7–1.0	0.15
Normal BMI		73	37.8	37	46.3			
Preobesity		54	28.0	22	27.5			
Obesity grade I		28	14.5	6	7.5			
Obesity grade II/III		23	11.9	7	8.8			
**Self-reported chronic health conditions**						nd		
Heart problems		8	4.2	5	6.3			
High blood pressure		40	20.7	20	25.0			
Lung problem		9	4.7	6	7.5			
Stomach ulcers		44	22.8	21	26.3			
Diabetes		11	5.7	7	8.8			
Liver problem		9	4.7	3	3.8			
Pneumonia		16	8.3	4	5.0			
Any cancer		6	3.1	1	1.3			
Arthritis		19	9.8	7	8.8			
Recent weight loss		28	14.5	15	18.8			
Recurring boils		9	4.7	2	2.5			
**Self-reported health conditions (5 days preceding the interview)**						nd		
Chills		27	14.0	13	16.3			
Fever w/no chills		29	15.0	16	20.0			
Fatigue		93	48.2	39	48.8			
Flu-like illness		48	24.9	13	16.3			
Loss of appetite		53	27.5	27	33.8			
Coughing		51	26.4	18	22.5			
Back pain		61	31.6	27	33.8			
Stomach pain		79	40.9	41	51.3			
Vomiting		27	14.0	13	16.3			
Constipation		67	34.7	33	41.2			
Painful joints		60	31.1	26	32.5			

OR: odds ratio, not adjusted.

a OR per unit change in ordered categories – presented is the linear effect determined by Orthogonal Polynomial Coding.

b not determined.

c rainwater/pond: rain water run off collected in water pans, lagoons or sand dams.

Higher potential risk towards CRC was associated with living in rural areas than urban areas (unadjusted OR 1.6; 95%CI 1.0–2.7; P = 0.08) (Table 1). Human ownership or contact with livestock and several socio-economic indicators were associated with a higher risk of CRC but none were statistically significant.

Forty six percent of the cases had normal BMI but 27.0% were pre-obese (Table 1). Self-reported chronic health conditions were mainly stomach ulcers (26.3% cases: 22.8% controls) and high blood pressure (25.0% cases; 20.7% controls). The most recent co-morbidities or symptoms of infections were stomach pain (51.3% cases; controls 40.9%), fatigue (48.8% cases; 48.2% controls) and constipation (41.2% cases; 34.7% controls).

### 3.2 Association between fermented (FDP) and non-fermented dairy products and adenocarcinoma

In general, the consumption of tFDP was associated with slightly elevated but not statistically significant risks of CRC (OR 1.4; CI 0.7–2.7; P = 0.34) (Table 2). Starter culture made commercial sour milk (cFDP) (mala/lala) was not associated with an increased risk (OR 1.0; CI 0.6–1.7; P = 0.95) while yoghurt (yFDP) consumption showed a slightly but not significant decreased risk CRC (OR 0.8; CI 0.4–1.4; P = 0.47).

**Table 2:** Food consumption in relation to CRC among cases and controls in the study population.

N = 273	Controls		Cases		OR^A^	95% CI	P-value
	n = 193	%	n = 80	%			
Raw cow milk	5	2.6	3	3.8	1.5	0.3–6.9	0.62
Pasteurized cow milk	105	54.4	48	60.0	1.3	0.8–2.3	0.33
Pasteurized goat milk	4	2.1	4	5.0	2.5	0.6–11.6	0.23
Spontaneously FDP (tFDP)	36	18.7	19	23.8	1.4	0.7–2.7	0.34
tFDP1	6	3.1	4	5.0	1.9	0.5–7.6	0.33
tFDP2	20	10.3	9	11.3	1.1	0.5–2.7	0.78
Both tFDP1 and tFDP2	10	5.1	6	7.5	1.5	0.5–4.7	0.42
Starter culture sour milk (mala/lala) (cFDP)	77	39.9	31	38.8	1.0	0.6–1.7	0.95
Yoghurt intake (yFDP)	93	48.2	29	36.3	0.8	0.5–1.4	0.47
Yoghurt sweetened^a^	46	23.8	16	20.0	0.8	0.4–1.7	0.63
Yoghurt with fruit	28	14.5	4	5.0	0.4	0.1–1.4	0.17
Yoghurt natural	7	3.6	3	3.8	1.0	0.2–4.2	0.98
yFDP type combinations	12	6.2	4	7.5	1.2	0.4–3.4	0.76
Butter	4	2.1	3	3.8	2.1	0.4–10.6	0.34
Tea with milk	153	79.3	71	88.8	2.1	1.0–4.8	0.06
Tea w/no milk	45	23.3	15	18.8	0.7	0.3–1.4	0.28
Coffee with milk	39	20.2	21	26.3	1.8	1.0–3.5	0.07
Coffee w/no milk	30	15.5	12	15.0	1.2	0.6–2.6	0.61
Porridge WFM^b^	16	8.3	5	6.3	0.6	0.2–1.8	0.36
Porridge WNM^c^	51	26.4	25	31.3	1.1	0.6–2.0	0.81

OR^A^: adjusted odds ratio.

a Yoghurt with added sweeteners and flavours.

b WMF: porridge with fermented milk. Fermented milk was added into the porridge during preparation.

c WNM: porridge without fermented milk. Raw milk was added into the porridge during preparation.

Consumption of cereal-based porridge containing cFDP or yFDP was not associated with an increased risk (OR 0.6; CI 0.2–1.8; P = 0.36). Adding plain milk to tea or coffee showed slightly elevated risks for CRC (OR 2.1; CI 1.0-4.8; P = 0.06) whereas tea without milk (OR 0.7; CI 0.3–1.4; P = 0.28) and coffee without milk (OR 1.2; CI 0.6–2.6; P = 0.61) did not. The values did not change noteworthy when other case definitions were applied (Fig. A.1 in the Supplementary material). We assume that the load of viable SBSEC reaching the intestinal tract can be neglected in all the consumed products except for tFDP, since raw milk, the assumed vector of SBSEC, did undergo at least one heat treatment step during the production process or the preparation process at home level.

### 3.3 Association of consumption of other foods with CRC

All sausages types were associated with increased risk of CRC; pork sausages (OR 2.5; CI 1.2–5.1; P = 0.01), chicken sausages (OR 2.6; CI 0.9–8.0; P = 0.09) and beef sausages (OR 1.9; CI 1.1–3.4; P = 0.03) (Table A.1 in the Supplementary material). In addition, poultry (OR 1.9), processed sandwich meat (OR 2.1), roasted red beef (OR 1.7) and unroasted red meet (OR 1.3) were associated with CRC, however, not reaching significance levels. All other food products such as bacon, fish and canned beef, beans, lentils, green grams and peas showed no noteworthy association.

### 3.4 Association of lifestyle characteristics with CRC

Alcohol consumption for a period of more than nine years was not associated to CRC likely due to low number of corresponding study participants (controls 13/193; cases 8/80) (Table A.2 in the Supplementary material). However, alcohol consumption showed an increased risk for CRC, which was statistically significant higher in participants that reported former alcohol consumption (OR 2.2; CI 1.2–4.0; P = 0.02) or ever consumed alcohol (OR 2.2; CI 1.2–4.2; P = 0.01). Similarly, smoking showed slightly increased risks of CRC but this association was not statistically significant even for participants who have smoked for more than seven years. There was no evidence that physical activities were associated with CRC (Table A.2 in the Supplementary material).

### 3.5 Prevalence of *Sii/SBSEC* and co-isolated non-SBSEC bacteria among clinical samples and participants

From the overall 413 participants only 196 provided clinical samples comprised of 196 rectal swabs samples, 163 blood samples, 23 faecal and 4 polyp biopsy samples. Clinical sample types were obtained only once per participant. None of the four polyp biopsy samples yielded presumptive *Sii*/SBSEC isolates while no bacterial colonies were observed from the 163 blood samples. From the rectal swabs and faecal samples, 1386 presumptive SBSEC isolates were obtained. Of these, 1053 and 333 were obtained from MS Agar and KFS Agar, respectively. DNA of all 1386 rectal swab and faecal isolates was amplified using a rep-PCR assay. Clustering by sample and rep-fingerprint to obtain one representative isolate per fingerprint per sample yielded 812 unique isolate DNA samples out of the total of 1386 isolates for further processing by the SBSEC-specific PCR assay. Fifty-nine out of the 812 were confirmed SBSEC isolates. Using the rep-fingerprint clustering to recalculate overall prevalence, 95 out of 1386 (6.9%) isolates were assigned to the SBSEC. For subspecies identification, partial sequencing of the *groEL* gene was performed to identify the 95 isolates identified as SBSEC members. Via rep-fingerprint linkage, *groEL* sequencing yielded 72 *Sii*, 6 *S. lutetiensis*, 1 *Sgm*, 12 *Sgp* and 4 untyped SBSEC members. *Sgg* and *S. equinus* were not isolated. The 95 SBSEC isolates were obtained from 46 out of 196 participants that provided clinical samples. This resulted in an overall SBSEC patient prevalence of 23.5%. Out of these patients, 31 were determined positive only for *Sii*, 7 only for *Sgp*, 2 only for *S. lutetiensis* and 1 for a not further identified SBSEC isolate. Combined carriage of SBSEC species was detected in 1 patient each for *Sii*&*Sgm*, *Sii*&*Sgp* and *S. lutetiensis*&*Sgp*. Two patients were determined to carry *Sii*&*S. lutetiensis.*

A limited selection of isolates (n = 30 out of 1356) from highly prevalent rep-PCR fingerprints of isolates not identified as SBSEC member were subjected to partial 16S rRNA gene sequencing. The isolates originated from 25 out of 196 participants. Half of the 30 strains were identified as *Enterococcus faecalis,* four *Streptococcus salivarius* and two *Enterococcus durans.* Out of the remaining nine isolates, five could not be identified while single isolates of *Cyanobacteria* spp, *Lactococcus lactis*, *Leuconostoc lactis* and *Streptococcus agalactiae* were described.

### 3.6 Prevalence of *Sii* stratified by colon health condition and association of faecal carriage by consumption of dairy products

**Fig. 2:**
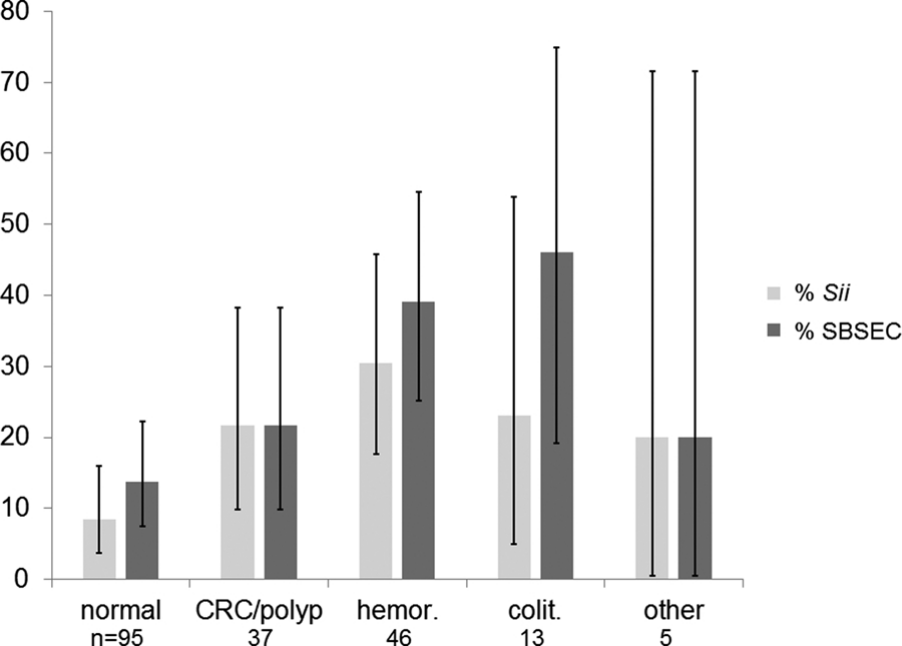
Faecal carriage of SBSEC (dark grey) and *Sii* (light grey) in persons with CRC and those with normal colon, haemorrhoids, colitis and other conditions. Error bars indicate 95% confidence interval (CI) boundaries.

The prevalence of *Sii* was 17.3% (34 out of 196 participants). The observed prevalence of *Sii* was significantly lower in the control group (8.4%, vs 21.6%: OR: 4.6; CI 1.3–15.9) indicating that *Sii* is associated with CRC. Interestingly, the highest prevalence of 30.4% (n: 46; CI: 17.7%-45.8%) was observed in participants diagnosed with haemorrhoids (Fig. 2). Similarly, prevalence of SBSEC carriage was lower among controls compared to cases (13.7 vs 21.6%; OR: 2.1; CI: 0.7–6.3) but the association was not statistically significant. Again SBSEC prevalence was highest for haemorrhoids 39.1% (18/46).

Among the 196 persons that contributed a clinical sample, the persons who consumed tFDP (14.3%; n = 49) featured a lower *Sii* carriage prevalence compared to the non-consumers (14.3% vs. 18.4%; OR: 0.7; CI 0.3–1.8). Consumers of yoghurt (yFDP) had *Sii* detected less often (13.6%; n = 103) than participants that did not consume yFDP (21.5%; n = 93). cFDP consumers had a carriage rate of 11.5% (10/87) in contrast to 22.0% (24/109) among non-consumers of cFDP. Separate calculations for exclusive tFDP consumers without yFDP or cFPD consumption were not performed due to a sample number of only 8 patients that would not yield meaningful conclusions.

## 4 Discussion

The determination of exposure and outcome information as well as control of confounders represent important steps in establishing a suspected epidemiological link between potential risk factors and cancer (Susser, 1973). tFDP in several sub-Saharan countries contain high levels of live *Sii* (Abdelgadir et al., 2008, Jans et al., 2012a, Jans et al., 2013b, Wullschleger et al., 2013). Recently, a 9.4% isolate prevalence of *Sii* was also reported in artisanal cheese from North Eastern Brazil (Medeiros et al., 2016) as well as tFDP in Bangladesh (Jans et al., 2016, Rashid et al., 2007) indicating the relevance of the findings of this study beyond Africa (Jans et al., 2015). The association of faecal carriage of *Sii* and consumption of tFDP and CRC had not been previously reported in the literature. Therefore, our study elucidated the link between exposures such as consumption of tFDP and other food groups, *Sii* faecal carriage, smoking, drinking of alcohol, engaging in physical activity as well as contact with livestock and the presence of CRC.

In this study, the proportion of males to females in the control group, was inversely proportional to the community level of Kenya’s national distribution (51% females; 49% males) (Kenya National Bureau of Statistics, 2010). However, the case group had a significant higher number of males than women, which can be expected for CRC (International Agency for Research on Cancer, 2012). This study also confirmed that the association of age above 50 years was a risk factor to CRC (Howlader et al., 2015), even though the age group of 40–49 years was also at risk of CRC. Thereby, the logistic regression models were adjusted for age, gender and rural residence.

In this study, *Sii* was elevated among persons with CRC when compared to persons with normal colon (21.6%:8.4%) showed similar associations for *Sii* to CRC as to those of other SBSEC members (Ben-Chetrit et al., 2017). The implied health risk of this *Sii* carriage in relation to CRC will require further investigations to elucidate causality of SBSEC and *Sii* in CRC as well as functional and genomic comparisons between the different *Sii* lineages. So far, little evidence on *Sii*-related diseases is available as mostly *S. lutetiensis* was detected or if *S. infantarius*-specific disease associations were reported then without differentiation between *Sii* and *S. lutetiensis* (Ben-Chetrit et al., 2017, Corredoira et al., 2013). Previous studies implicating SBSEC or *S. infantarius* in human infections might therefore require additional follow up work to elucidate the epidemiologic and functional data under the more discriminative current SBSEC taxonomy (Ben-Chetrit et al., 2017, Jans et al., 2016, Jans et al., 2015). Furthermore, larger study settings in hospitals and among the general population will be needed for enhanced insights into the epidemiology and disease associations of the SBSEC subspecies.

To estimate the public health risk posed by *Sii*, it remains to be investigated whether *Sii* possesses proliferative advantage during colonic malignancies as reported for *Sgg* (Tjalsma et al., 2012). The prevalence of colonic malignancy in this study was higher (29.3%; 80 out of 273) than 18.9% previously reported from a similar study setup in France (Chirouze et al., 2013). This could in part be related to different hospital populations and particularly low health care insurance coverage in Kenya (Kazungu and Barasa, 2017). This low coverage could lead people to only seek care when a disease is already progressed far and would thus yield a more pronounced stage of malignancy development. However, colon malignancy among both hospital populations was still significantly lower than that determined for IE patients of up to 69% (Olmos et al., 2016) and 84.6% (Alozie et al., 2015).

The prevalence reported for *Sii* in this study was similar to 11% *Sii* prevalence determined for rectal swabs of colonoscopy patients in Brazil using a qPCR assay (Lopes et al., 2014). In contrast, general SBSEC prevalence in France was reported at 6% for normal colonoscopy (Chirouze et al., 2013). In Taiwan, *Sgp* was predominant at 47–58% among a colorectal screening population using a blood-agar and API-based typing approach, while *S. infantarius* branch members (*S. lutetiensis* and *Sii*) were the second most predominant SBSEC group at 36–42% (Huang et al., 2008). Sequencing-based comparison of the gut microbiota composition of Ghanaian and Dutch infants also revealed different relative abundances of SBSEC members by geography and vaccination response (Harris et al., 2017). Thus, overall SBSEC and SBSEC species prevalence seems to differ by geography as previously reported for gut microbiota composition (Yatsunenko et al., 2012). Furthermore, isolation and identification methodology as well as differences in study population composition among these hospital-based studies might contribute to the observed differences. Particularly for this study, the possible consumption of *Sii* via tFDP should not be neglected.

This study is the first assessment of *Sii* in relation to food consumption, human diseases and carriage. In the presented study, we found no evidence that FDP consumption increases the risk of CRC. It seems that products such as yoghurt (yFDP) and cultured sour milk (mala/lala) (cFDP), originating from industrial processes, likely with ‘good manufacturing practices’ concepts implemented, had inverse or slight inverse association with CRC or faecal carriage of *Sii*. Out of these FDP, only tFDP is expected to contain *Sii* but not yFDP or cFDP (Jans et al., 2017). It remains to be investigated whether the obtained human *Sii* in this study are related to the dairy adapted African variants present in sub-Saharan Africa or rather represent commensal lineages (Jans et al., 2016). However, the presence of *Sii* also in non-tFDP consumers suggests at least a secondary source from the environment, animals or simply indicates commensal status of certain *Sii* in the gastrointestinal tract of humans.

Foods that were significantly associated with CRC in our study, were eggs, pork sausages and beef salami. While the association of processed red meat products with increased risk of CRC has been established (Santarelli et al., 2008), the case for eggs is less clear. Previous studies have suggested the association of CRC with high cholesterol and possibly egg consumption (Jarvinen et al., 2001, Lee et al., 2009, Steinmetz and Potter, 1994), evidence is however still incomplete and will require further research.

Other foods with potential risk towards CRC were chicken sausages, poultry with skin, processed meat, pork and roast beef. Multiple studies associated consumption of red meat with an increased risk of colorectal adenomas and cancers (Joint WHO/FAO expert consultation group, 2003 Murdoch et al., 2009). However, it is still unclear whether this effect was attributable to substances in red meat or changes resulting during cooking and processing (Joint WHO/FAO expert consultation group, 2003).

This study identified alcohol consumption as a major risk factor of developing CRC. Consumption of alcohol, cigarette smoking (Erhardt et al., 2002, IARC Working Group on the Evaluation of Carcinogenic Risks to Humans, 1988, Jedrychowski et al., 2002, Pflaum et al., 2016) and lack of adequate levels of physical activity have been previously reported to be associated with CRC (International Agency for Research on Cancer and world health organization, 2002, World Health Organization, 2002). CRC association with alcohol intake is concentrated mainly on persons that had low folate intake (Joint WHO/FAO expert consultation group, 2003). Also drinking small amounts of alcohol may not increase the risk of developing CRC (World Health Organization, 2002). However, other studies indicated that smoking and alcohol use leads to increased oxidative stress that subsequently leads to DNA damage and consequently lead to CRC (Valko et al., 2004).

This study did not reveal a single blood isolate of *Sii* or other SBSEC members from the collected blood samples. Participants were not hospitalized for infective endocarditis or bacteraemia. In contrast, many other studies that examined participants by colonoscopy primarily recruited among bacteraemia and infective endocarditis patients. Among these endocarditis patients, SBSEC members were identified from only 6% of all positive blood cultures assays (Alozie et al., 2015, Chirouze et al., 2013, Corredoira et al., 2015, Olmos et al., 2016). Therefore, the absence of SBSEC members from blood cultures of participants enrolled in this study meets the expectations.

The Mitis Salivarius agar used in this study allowed for a semi-selective isolation of *Sii* as the main study objective. Preliminary tests using SBSEC type strains of *Sii, S. lutetiensis, Sgg, Sgp, Sgm, S. equinus, S. bovis* and *S. alactolyticus* as well as over 30 *Sii* and *S. lutetiensis* strains from human and tFDP sources allowed cultivation of all strains except *S. alactolyticus* DSM 20728^T^ under the applied conditions on MS agar (data not shown). Therefore, the probability of false negative outcomes is expected to be minimal for *Sii, S. lutetiensis, Sgm*, *Sgg*, *Sgp* and *S. bovis/S. equinus*.

## 5 Limitations

This study had the following limitations: the study was designed as a case-control study based at the health-facility and therefore recruited participants may not represent the overall population of the country or a certain geographic location of the country. The participant selection was potentially skewed towards certain colonic health conditions.

The study relied on histology examination of colon tissue samples for diagnosis of cancer state. Histology examination returned the status for tumour (carcinoma) and polyp. However, colorectal polyps were not differentiated further between non-neoplastic and neoplastic types. Non-neoplastic polyps have no malignant potential, which includes benign lesions (hyperplastic polyps). Neoplastic polyps include pre-malignant (adenoma) or malignant (carcinoma) types of which the later was determined by histologists as tumour (carcinoma). Available patient data from KNH do not allow separating the study population into non-neoplastic and neoplastic polyp types. Therefore, a mixed health status concerning non-neoplastic (benign) and neoplastic (adenoma) polyp types was obtained for all participants classified as polyp carriers in this study. Alternative case definitions were defined to perform corresponding sensitivity analyses.

The usual intake of various foods including FDP was assessed by food frequency questionnaires. Thereby, data on quantification of portion sizes or daily intakes was limiting. However, such questionnaires were successfully employed by others suggesting their applicability to the settings in this study (Haftenberger et al., 2010, Tantamango et al., 2011).

## 6 Conclusions

Our study indicated no increase in risk towards CRC through tFDP consumption but an association of *Sii* with CRC. Interestingly, consumption of tFDP and other FDPs lead to a slight decrease in faecal carriage of *Sii.* Unexpectedly, this study also revealed an elevated faecal carriage of *Sii* in persons with haemorrhoids, which requires further investigations. In addition, the rural population is at greater risk towards CRC than urban dwellers. This might not be explained within the scope of this study, even though there is higher proportion of the over-50-year-old persons living in the rural regions.

The presence of *Sii* in many tFDP consuming and non-consuming participants without CRC or colon disorders suggests a potential commensalism of *Sii* in humans. However, due to the CRC association, the novel finding of haemorrhoid association and still unknown pathogenicity factors potentially shared between *Sii* and other SBSEC members, further genetic and functional characterization of the different strains and phylogenetic *Sii* lineages as well as epidemiologic data will be required to formulate appropriate and sustainable interventions. These interventions will target the millions of tFDP consumers in sub-Saharan Africa and in other regions of the world where *Sii* is present in tFDP and will therefore need to be based on strong scientific evidence.

## Conflict of interest

The authors declare no conflict of interests.
