# Supplementary material for: Investigating the association between African spontaneously fermented dairy products, faecal carriage of Streptococcus infantarius subsp. infantarius and colorectal adenocarcinoma in Kenya
Source: Acta Trop. 2018 Feb;178:10–8. doi: 10.1016/j.actatropica.2017.10.018 (PMC5766739; doi:10.1016/j.actatropica.2017.10.018)

		CD1		Alt.CD1		Alt.CD2	
Case def.:		Norm/CRC		Norm/colon		N+C/CRC	
		contr.	case	contr.	case	contr.	case
Raw cow milk	%	3%	4%	3%	4%	3%	4%
	N_{pos}/N_{tot}	5/193	3/80	5/193	8/220	10/333	3/80
Spontan. FDP	%	19%	24%	19%	25%	22%	24%
(tFDP)	N_{pos}/N_{tot}	36/193	19/80	36/193	56/220	73/333	19/80
Mala	%	40%	39%	40%	36%	38%	39%
(cFDP)	N_{pos}/N_{tot}	77/193	31/80	77/193	79/220	125/333	31/80
Yoghurt	%	48%	36%	48%	45%	49%	36%
(cFDP)	N_{pos}/N_{tot}	93/193	29/80	93/193	98/220	162/333	29/80

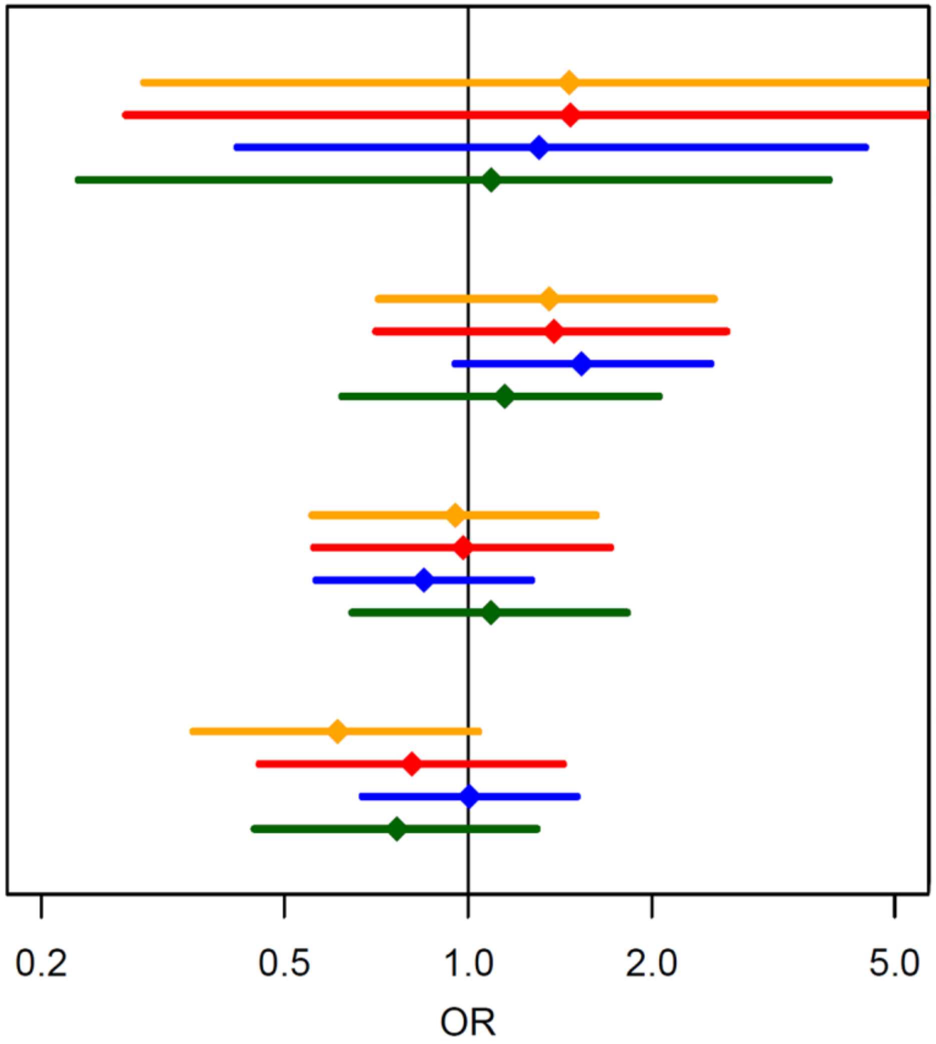

Fig. A.1:

Sensitivity analysis assessing the robustness of the results with respect to alternative case definitions. Presented are odds ratios and their corresponding confidence intervals estimated by logistic regression:
orange (primary definition, unadjusted) Cases: CRC, polyps – Controls: normal gastrointestinal tract (GIT); **red** (primary definition, adjusted for age, sex and residency); **blue** (alternative definition 1, adjusted) Cases: CRC, polyps, haemorrhoids, colitis – Controls: normal GIT; **green** (alternative definition 2, adjusted) Cases: CRC, polyps – Controls: normal GIT, haemorrhoids, colitis.

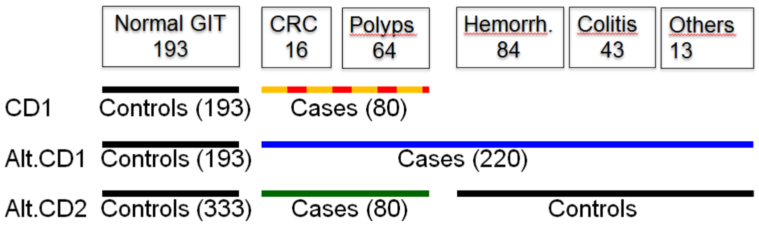

Supplement: Supplementary file 1 [file mmc1.pdf]
