# Supplementary material for: Investigating the association between African spontaneously fermented dairy products, faecal carriage of Streptococcus infantarius subsp. infantarius and colorectal adenocarcinoma in Kenya
Source: Acta Trop. 2018 Feb;178:10–8. doi: 10.1016/j.actatropica.2017.10.018 (PMC5766739; doi:10.1016/j.actatropica.2017.10.018)
Supplement: Supplementary file 2 [file mmc2.docx]

Table A.1: Association of consumption of other non-dairy food products with CRC.

|  | **N=273** | **Control** | | **Cases** | | **OR^A^** |  | **P-value** |
| --- | --- | --- | --- | --- | --- | --- | --- | --- |
|  |  | **n=193** | **%** | **n=80** | **%** |  | **95% CI** |  |
|  | Eggs | 153 | 79.3 | 68 | 85.0 | 1.6 | 0.8-3.3 | 0.20 |
|  | Pork sausages | 23 | 11.9 | 18 | 22.5 | 2.5 | 1.2-5.1 | 0.01 |
|  | Beef sausages | 60 | 31.1 | 32 | 40.0 | 1.9 | 1.1-3.4 | 0.03 |
|  | Chicken sausages | 8 | 4.2 | 7 | 8.8 | 2.6 | 0.9-8.0 | 0.09 |
|  | Poultry | 139 | 72.0 | 65 | 81.3 | 1.9 | 1.0-3.8 | 0.06 |
|  | Bacon | 7 | 3.6 | 3 | 3.8 | 0.8 | 0.2-3.4 | 0.78 |
|  | Sandwich meat | 8 | 4.2 | 5 | 6.3 | 2.1 | 0.7-7.1 | 0.21 |
|  | Processed meat | 9 | 4.7 | 6 | 7.5 | 2.3 | 0.7-7.1 | 0.15 |
|  | Canned beef | 12 | 6.2 | 5 | 6.2 | 1.0 | 0.4-3.4 | 0.88 |
|  | Hamburger | 4 | 2.1 | 2 | 2.5 | 0.9 | 0.1-5.6 | 0.92 |
|  | Pork | 21 | 10.9 | 13 | 16.3 | 1.4 | 0.6-3.2 | 0.38 |
|  | Roasted beef | 62 | 32.1 | 33 | 41.3 | 1.7 | 1.0-3.1 | 0.07 |
|  | Red meat unroasted | 141 | 73.1 | 61 | 76.3 | 1.3 | 0.7-2.4 | 0.44 |
|  | Fish | 105 | 54.4 | 45 | 54.9 | 1.0 | 0.6-1.7 | 0.94 |
|  | Beans | 135 | 70.0 | 59 | 73.8 | 1.1 | 0.6-2.0 | 0.80 |
|  | Lentils | 46 | 23.8 | 18 | 22.5 | 1.0 | 0.5-1.9 | 0.94 |
|  | Green grams | 133 | 68.9 | 56 | 68.8 | 1.0 | 0.5-1.7 | 0.90 |
|  | Peas | 84 | 43.5 | 38 | 47.5 | 1.1 | 0.6-1.9 | 0.79 |
|  | Soy beans | 15 | 7.8 | 12 | 15.0 | 1.9 | 0.8-4.3 | 0.15 |

OR^A^: adjusted odds ration; nd: not done.
