# Supplementary material for: Investigating the association between African spontaneously fermented dairy products, faecal carriage of Streptococcus infantarius subsp. infantarius and colorectal adenocarcinoma in Kenya
Source: Acta Trop. 2018 Feb;178:10–8. doi: 10.1016/j.actatropica.2017.10.018 (PMC5766739; doi:10.1016/j.actatropica.2017.10.018)
Supplement: Supplementary file 3 [file mmc3.docx]

Table A.2: Lifestyle characteristics as association with CRC

|  | **N=273** | **Controls** | | **Cases** | | **OR^A^** |  |  |
| --- | --- | --- | --- | --- | --- | --- | --- | --- |
|  |  | **n=193** | **%** | **n=80** | **%** |  | **95% CI** | **P** |
| **Tobacco smoking** | |  |  |  |  |  |  |  |
|  | Ever smoked | 41 | 21.2 | 27 | 33.8 | 1.6 | 0.8-3.3 | 0.19 |
|  | Current smoker | 5 | 2.6 | 3 | 3.8 | 1.5 | 0.3-6.6 | 0.62 |
|  | Former smoker | 37 | 19.2 | 24 | 30.0 | 1.5 | 0.7-3.1 | 0.27 |
|  | Smoking > 7 yrs | 27 | 14.0 | 15 | 19.0 | 1.0 | 0.4-2.3 | 0.97 |
| **Alcohol consumption** | |  |  |  |  |  |  |  |
|  | Ever used alcohol | 70 | 36.3 | 44 | 55.0 | 2.2 | 1.2-4.2 | 0.01 |
|  | Current alcohol user | 15 | 7.8 | 6 | 7.5 | 1.2 | 0.4-3.3 | 0.77 |
|  | Former alcohol user | 62 | 32.1 | 41 | 51.3 | 2.2 | 1.2-4.0 | 0.02 |
|  | Alcohol >9yrs | 13 | 6.7 | 8 | 10.0 | 1.2 | 0.4-3.2 | 0.78 |
| **Physical activity** | |  |  |  |  |  |  |  |
| **Doing vigorous physical activities** | |  |  |  |  |  |  |  |
|  | Rarely/ never | 177 | 91.7 | 76 | 95.0 | - |  |  |
|  | yes | 16 | 8.3 | 4 | 5.0 | 0.9 | 0.3-2.9 | 0.86 |

OR^A^: adjusted odds ratio; -: Reference point/indicator.
